# Palliative Care Interventions Among Hispanic Inner-city Patients with Advanced Liver Disease

**DOI:** 10.1093/geroni/igaf122.2617

**Published:** 2025-12-31

**Authors:** Zachary Song, Mekiayla (Meki) Singleton, Carin van Zyl, Aaron Storms, Susan Enguidanos

**Affiliations:** University of Southern California Leonard Davis School of Gerontology, Los Angeles, California, United States; Rutgers University, Newark, New Jersey, United States; LA General Medical Center, Los Angeles, California, United States; Los Angeles General Medical Center, Los Angeles, California, United States; University of Southern California, Los Angeles, California, United States

## Abstract

Advanced liver disease is the 9th leading cause of death in the United States and the 7th leading cause of death among Hispanic individuals. Patients suffering from end-stage liver disease frequently encounter various physical and psychosocial stressors, yet routine access to palliative care is limited. This study aimed to identify the primary needs of a predominantly Hispanic patient population with advanced liver disease and the interventions provided by a palliative care team within a hepatology clinic at a large urban public hospital. We conducted a retrospective qualitative analysis of palliative care team medical record entries from a pilot palliative care program. Participants with advanced liver disease (MELD ≥20) were recruited between 2016 and 2017. Thematic analysis was performed to identify key patient stressors, sources of patient support, and interventions conducted by the palliative care team. Of the 65 participants, 94% identified as Hispanic, with over half undocumented. Common patient stressors included physical symptoms, financial hardship, and challenges related to immigration. Family involvement and religious faith were primary sources of support. Palliative care interventions included psychosocial counseling, end-of-life care planning, social service and public benefit referrals (e.g., food, in-home support, and disability insurance), substance use management, and help navigating the healthcare system. Findings illustrate the complex interplay between social determinants of health and disease management for Hispanic patients with advanced liver disease. Integrating palliative care into hepatology clinics addresses these multifaceted needs by providing tailored psychosocial and medical support. Further research is needed to optimize and expand these interventions.

